# An Unusual Observation in Metastatic Neuroendocrine Neoplasm: Diffuse Pattern Hepatic [ ^68^ Ga]Ga-DOTATATE Uptake Related to Micro-metastatic Disease and Discordance between Dual-Tracer PET-CT Findings and MIB-1 Labelling Index

**DOI:** 10.1055/s-0044-1801384

**Published:** 2025-01-08

**Authors:** Parth Baberwal, Sunita N. Sonavane, Sandip Basu

**Affiliations:** 1 Radiation Medicine Centre, Bhabha Atomic Research Centre, Tata Memorial Hospital Annexe, Mumbai, Maharashtra, India; 2 Homi Bhabha National Institute, Mumbai, Maharashtra, India

**Keywords:** metastasis, NET, DOTATATE, FDG, PET, discordance, de-differentiation, dual-tracer PET

## Abstract

Neuroendocrine neoplasms (NENs) are a rare and diverse group of neoplasms that can originate from neuroendocrine cells in any organ. We herein present a patient with Grade II neuroendocrine tumor (NET) of the pancreas with bilobar liver metastasis and a MIB-1 labelling index of 15%, who underwent various systemic and targeted therapies. On follow-up, dual-tracer PET-CT imaging with [ ^68^ Ga]Ga-DOTATATE PET/CT showed new onset skeletal metastases and diffuse pattern SSTR (somatostatin receptor) expression in the left lobe of the liver (Krenning score 3), contrasted by absent uptake on [ ^18^ F]FDG. Magnetic resonance imaging of the liver confirmed sub-centimetric left liver lobe lesions, further biopsy of which suggested Grade-III NET exhibiting high Ki-67 (55–60%). Thus, a discordance was observed between Ki-67 and the dual-tracer PET-CT findings, emphasizing the complexity of NEN imaging (with possibility of differentiation even in a relatively high Ki-67) and the importance of using multiple tracers for accurate assessment in guiding evidence-based management strategy.

## Introduction

Neuroendocrine neoplasms (NENs) are a varied set of tumors that develop from neuroendocrine cells and have different clinical outcomes and presentations. 1 Functional imaging is a pivotal component for diagnosing, staging, and managing neuroendocrine tumors (NETs). 2 Using dual-tracer positron emission tomography/computed tomography (PET/CT) imaging with [ ^68^ Ga]Ga-DOTATATE and [ ^18^ F]FDG is especially useful for identifying different characteristics of tumors and guide treatment approaches. 3 This report details a patient with Grade II NET with 15% MIB-1 labeling index at presentation, who initially underwent various systemic and targeted therapies. Follow-up revealed disease progression and inverse discordance between pathology and dual-tracer PET-CT imaging, highlighting the complexity of NET imaging and the necessity of using multiple tracers for accurate assessment in correlation with detailed histopathology, which is crucial for guiding an evidence-based management strategy.

## Case Report

A 42-year-old male with a history of diabetes and a family history of undiagnosed malignancy in his mother and grandmother presented with abdominal pain. Diagnostic evaluation revealed a lesion in the tail of the pancreas with multiple bilobar liver metastases. A biopsy of a liver lesion confirmed a Grade II NET with an MIB-1 labeling index of 15%. Dual-tracer PET-CT imaging was performed, [ ^68^ Ga]Ga-DOTATATE-PET/CT showed a somatostatin receptor (SSTR)-expressing lesion in the tail of pancreas (SUVmax: 8.45) and multiple SSTR-expressing bilobar liver lesions (SUVmax: 12.38). [ ^18^ F]FDG-PET/CT for further disease characterization demonstrated multiple hypodense liver lesions, few of them showing increased metabolism, largest in segment II, measuring 7.1 × 4.7 × 5.6 cm (SUVmax: 11.40), while the primary pancreatic lesion was ametabolic.

**Fig. 1:**
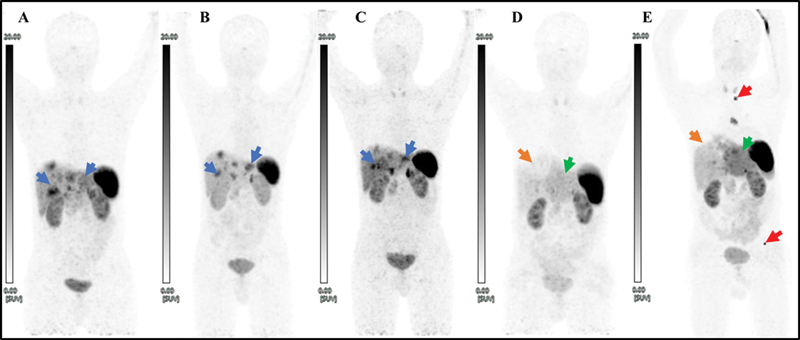
A series of maximum intensity projection (MIP) images of [ ^68^ Ga]Ga-DOTATATE PET/CT scan done at baseline ( **A** ), post 4 cycles of PRRT and 12 cycles of CAPTEM (4 prior to PRRT and 8 cycles sandwiched with PRRT) ( **B** ), post-CAPTEM and PRRT follow-up ( **C** ), post 5 cycles of TACE ( **D** ) and recent follow-up ( **E** ) showing bilobar liver metastasis (blue arrow) initially responding to systemic therapy; an area of photopenia in right upper aspect of liver post-TACE (orange arrow); heterogeneous diffuse left lobe tracer uptake (more than the normal uptake in the right lobe) that was observed in recent follow-up (E) and retrospectively noted in post-TACE scan (D) as well (green arrow); new-onset SSTR-expressing skeletal metastasis (red arrow) and solitary subcarinal lymphadenopathy. CAPTEM, capecitabine-temozolomide; PRRT, peptide receptor radionuclide therapy.

The patient underwent 4 cycles of peptide receptor radionuclide therapy ( Fig. 1A–C ), 12 cycles of capecitabine-temozolomide (CAPTEM), and 4 cycles of trans-arterial chemoembolization (TACE). At the end of treatment (EOT), serum chromogranin A levels were 3,064 mcg/L, and the patient reported clinical improvement. EOT [ ^68^ Ga]Ga-DOTATATE PET/CT indicated stable disease with TACE-related changes in the right lobe of the liver ( Fig. 1D ).

**Fig. 2:**
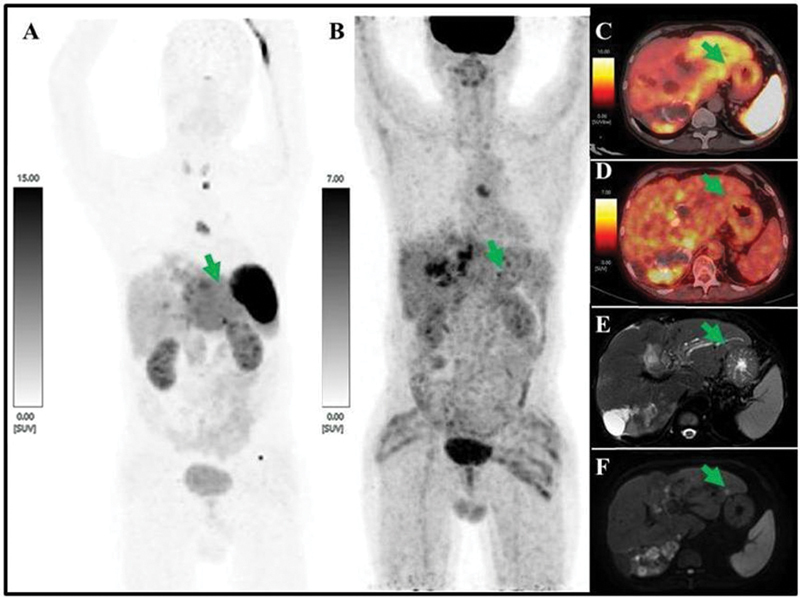
A MIP of [ ^68^ Ga]Ga-DOTATATE-PET/CT ( **A** ) showing heterogeneously diffuse tracer uptake in left lobe of liver (Krenning score 3) while MIP of [ ^18^ F]FDG-PET ( **B** ) is showing no correlative tracer uptake concentration. Fused axial view of [ ^68^ Ga]Ga-DOTATATE-PET/CT ( **C** ) and [ ^18^ F]FDG-PET/CT ( **D** ) showing similar findings as mentioned above. T2-weighted SPAIR (spectral attenuated inversion recovery) MRI axial view ( **E** ) showing T2 hyperintense enhancing multiple subcentimeter-sized left lobe of liver lesions and axial view of diffusion-weighted MRI ( **F** ) showing diffusion restriction in the same lesions (marked with green arrow). MIP, maximum intensity projection; MRI, magnetic resonance imaging.

At the last follow-up, approximately 1 year later, the patient reported new onset of abdominal and lower back pain. Follow-up dual-tracer PET-CT imaging revealed new SSTR-expressing bilobar liver lesions, SSTR-expressing subcarinal lymph nodes, and SSTR-expressing lytic lesions in the body of the D2 vertebra and left iliac bone ( Figs. 1E and 2A ). Additionally, there was diffusely increased SSTR expression (SUVmax: 9.2, Krenning score: 3) in the entire left lobe of the liver, with no definite focal uptake delineated on [ ^68^ Ga]Ga-DOTATATE ( Figs. 1E , 2A , and 2C ). [ ^18^ F]FDG-PET/CT revealed new hypermetabolic lesions in the right lobe of the liver (segment V/VI, measuring 3.9 × 1.6 cm with SUVmax of 7.70), a low metabolic subcarinal node (measuring 1.7 × 1.5 cm with SUVmax of 6.13), and minimal [ ^18^ F]FDG uptake in a few marrow lesions involving the D2 vertebra (SUVmax: 3.75) and left iliac bone ( Fig. 2B ). Magnetic resonance imaging (MRI) showed new T2-enhancing lesions in both liver lobes with diffusion restriction. The T2 hyperintense enhancing lesions in the left lobe of the liver were multiple and subcentimeter in size ( Fig. 2E, F ). Serum chromogranin A at this time was >9,000 mcg/L. A biopsy of the left lobe of liver revealed metastatic NET with a mitotic count of 12/10 hpf and no necrosis. Immunohistochemistry showed the tumor cells were diffusely positive for synaptophysin and chromogranin, negative for p40, and focally positive for somatostatin in approximately 5% of tumor cells. The MIB-1 labeling index was 55 to 60% in the most proliferative area. The patient is currently alive and continues to receive treatment from a multidisciplinary team for ongoing management.

## Discussion

Histologically, NENs are classified into well-differentiated NETs and poorly differentiated neuroendocrine carcinomas. 4 The MIB-1 index gauges the aggressiveness of tumors. 5 Elevated MIB-1 values reflect increased cell proliferation, suggesting a more aggressive tumor and a poorer prognosis. 6 The MIB-1/Ki-67 index continues to be fundamental in guiding the planning of oncologic therapies. 7 Tumors with higher proliferation indices necessitate more aggressive treatment approaches. 8 The most common site of NENs is the gastrointestinal tract, predominantly mid-gut, followed by lung. 9 10 NETs, on a molecular level, express SSTR, thus, targeted diagnostic and therapeutic approaches can be considered for management. NETs vary a lot in the extent and pattern of the metastasis. 11 In gastroenteropancreatic NETs, the most common site of metastasis is liver. 12 There have been reports of micro-metastatic pattern of disease in case of NETs. 13 14 D'Souza et al detected micro-metastatic disease initially with the help of SSTR-PET imaging, where it showed diffusely increased heterogeneous uptake which was more than splenic parenchyma. 14 Albeit liver concentrates [ ^68^ Ga]Ga-DOTATATE physiologically as well, in our case micro-metastases were suspected as left lobe of liver was concentrating [ ^68^ Ga]Ga-DOTATATE more intensely and diffusely than that of right lobe and uptake had increased significantly in comparison to previous PET/CT, and MRI showed several distinct sub-centimeter-sized metastatic liver lesions in the left lobe.

De-differentiation in NEN though an uncommon phenomenon is a possibility, wherein an increased aggressive profile in metastasis, especially in NETs, can be observed. 15 Less differentiated NENs demonstrate [ ^18^ F]FDG avidity secondary to high proliferative activity. 16 In our case, however, there was no significant focal/diffuse [ ^18^ F]FDG avidity in the left lobe of liver, while the histopathology report suggested grade of NET changed to grade III with the reported MIB-1 index increased to 55 to 60%. Thus, dual-tracer imaging with both SSTR- and [ ^18^ F]FDG PET/CT not only aided in visualizing lesions with different biology, but also harbored prognostic implications.

## Conclusion

We present a rare intriguing case of NET of tail of pancreas with liver, nodal, and skeletal metastasis post systemic and targeted therapies presenting with new-onset diffuse left lobe of liver SSTR expression on SSTR-based PET (and negligible uptake on FDG), which on biopsy suggested a relatively de-differentiated metastasis (initial MIB-1 of 15% progressing to 55–60%). This implies that the relation between MIB-1, de-differentiation, and [ ^68^ Ga]Ga-DOTATATE/[ ^18^ F]FDG avidity is yet to be completely explored.
